# Editorial: The Role of AAA+ Proteins in Protein Repair and Degradation

**DOI:** 10.3389/fmolb.2018.00085

**Published:** 2018-10-02

**Authors:** James Shorter, Walid A. Houry

**Affiliations:** ^1^Department of Biochemistry and Biophysics, Perelman School of Medicine at the University of Pennsylvania, Philadelphia, PA, United States; ^2^Department of Biochemistry, University of Toronto, Toronto, ON, Canada; ^3^Department of Chemistry, University of Toronto, Toronto, ON, Canada

**Keywords:** AAA+ ATPase, chaperone, protease, Hsp104, p97

ATPases Associated with diverse cellular Activities (AAA+) comprise a superfamily of proteins that perform a large variety of functions essential to cell physiology, including control of protein homeostasis, DNA replication, recombination, chromatin remodeling, ribosomal RNA processing, molecular targeting, organelle biogenesis, and membrane fusion (Hanson and Whiteheart, 2005; Erzberger and Berger, 2006; Snider et al., 2008). Members of this superfamily are defined by the presence of what is termed the AAA+ domain containing the canonical Walker A and B motifs required for ATP binding and hydrolysis (Hanson and Whiteheart, 2005). Typically, genomes encode approximately ten to several hundred AAA+ family members (Table 1; Finn et al., 2017), each of which is thought to be adapted to specific functional niches that necessitate precise mechanisms of substrate recognition and processing (Hanson and Whiteheart, 2005). The striking adaptive radiation of AAA+ proteins to operate in diverse settings illustrates the versatile utility of the AAA+ domain (Erzberger and Berger, 2006). AAA+ proteins typically form hexameric complexes and act as motors to remodel other proteins, DNA/RNA, or multicomponent complexes (Figure 1). Indeed, many chaperones and ATP-dependent proteases are or have subunits that belong to this superfamily (Figure 1; Olivares et al., 2016).

**Table 1:** Number of AAA+ proteins in model organisms^a^.

**Species**	**Number of proteins containing AAA+ domains**
*Arabidopsis thaliana* (Mouse-ear cress)	364
*Oryza sativa subsp. japonica* (Rice)	275
*Homo sapiens* (Human)	239
*Mus musculus* (Mouse)	135
*Danio rerio* (Zebrafish)	115
*Drosophila melanogaster* (Fruit fly)	98
*Caenorhabditis elegans* (Roundworm)	48
*Saccharomyces cerevisiae* (strain ATCC 204508/S288c) (Baker's yeast)	34
*Schizosaccharomyces pombe* (strain 972/ATCC 24843) (Fission yeast)	32
*Bacillus subtilis* (strain 168)	9
*Caulobacter crescentus*^b^ (strain NA1000/CB15N)	8
*Escherichia coli* (strain K12)	7

a *The table was obtained from the InerPro database (Finn et al., 2017)*.

b *Also known as Caulobacter vibrioides*.

**Figure 1:**
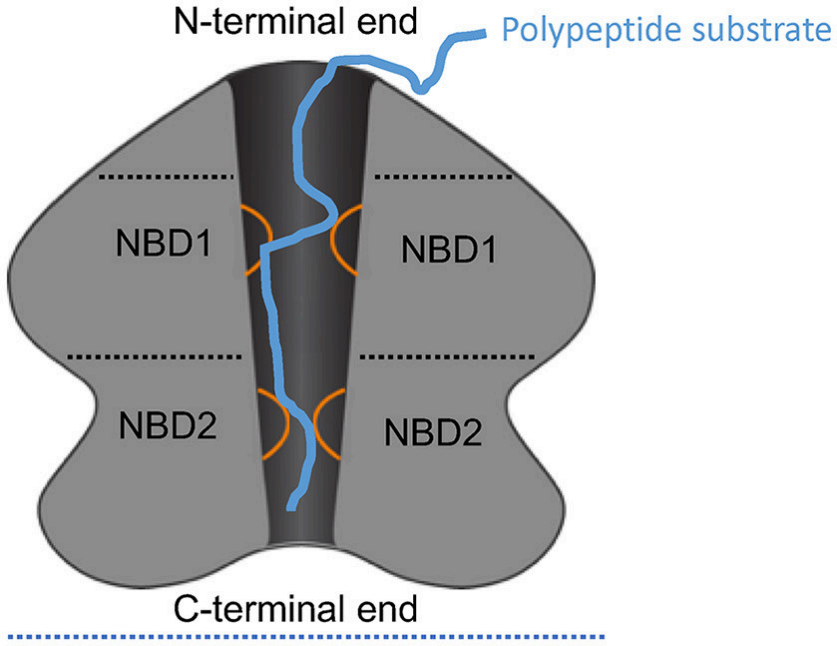
Schematic of ClpA as an example AAA+ hexamer. Schematic representation of the domain architecture and interactions of ClpA as an example AAA+ hexamer with two AAA+ domains per monomer. Here, a cutaway side view of the ClpA hexamer is shown to depict the central, polypeptide-conducting channel. ClpA contains three domains, including an N-terminal domain and two AAA+ domains: nucleotide-binding domain 1 and 2 (NBD1 and NBD2). The N-terminal domain interacts with regulators of substrate specificity, whereas the C-terminal end interacts with the chambered protease ClpP. Mobile loops from NBD1 and NBD2 (orange) project into the central channel and engage polypeptide substrate (blue) thereby enabling coupling of ATP hydrolysis to polypeptide translocation across the central channel.

Over recent years, there has been substantial progress in identifying the structure and functional mechanism of a large number of AAA+ proteins (Gates et al., 2017; Puchades et al., 2017; Ripstein et al., 2017; Zehr et al., 2017). In this research topic, several elements of this exciting progress are conveyed in 21 articles, which encompass a detailed structural and mechanistic view of several AAA+ chaperones and proteases, including: ClpX (Alhuwaider and Dougan; Bittner et al.; Elsholz et al.; LaBreck et al.; Vass et al.), ClpA (Bittner et al.; Duran et al.), ClpB and Hsp104 (Chang et al.; Duran et al.; Franke et al.; Johnston et al.), Hsp78 (Abrahão et al.), ClpC (Alhuwaider and Dougan; Elsholz et al.), ClpE (Elsholz et al.), Pontin (Mao and Houry), Reptin (Mao and Houry), FtsH (Alhuwaider and Dougan), 19S proteasome (Snoberger et al.; Yedidi et al.), Lon (Alhuwaider and Dougan; Bittner et al.; Fishovitz et al.), p97 (Hänzelmann and Schindelin; Saffert et al.; Ye et al.), Pex1/6 (Saffert et al.), CbbQ (Mueller-Cajar), rubisco activase (Bhat et al.), torsins (Chase et al.), and mitochondrial AAA+ proteases (Glynn). Here, we introduce these fascinating works.

## Studies on ClpXP, Lon, and related ATP-dependent proteases

In their research article, “The Protein Chaperone ClpX Targets Native and Non-native Aggregated Substrates for Remodeling, Disassembly, and Degradation with ClpP,” LaBreck et al. perform a series of elegant experiments to establish that ClpX possesses disaggregase activity against polypeptides that contain specific ClpX-recognition signals (LaBreck et al.). In the presence of ClpP, ClpX couples disaggregation of these substrates to their degradation. Importantly, they also establish that ClpXP prevents the accumulation of aggregates formed by proteins bearing ClpX recognition signals *in vivo* (LaBreck et al.). These studies illuminate ClpX as a protein disaggregase, which was previously underappreciated.

In their research article, “The Essential Role of ClpXP in *Caulobacter crescentus* Requires Species Constrained Substrate Specificity,” Vass et al. explore species-specific functions of ClpX (Vass et al.). Curiously, ClpX is essential in some species such as *C. crescentus*, but not essential in other bacteria such as *E. coli* (Vass et al.). Importantly, *E. coli* ClpX was unable to complement *C. crescentus* ClpX *in vivo* (Vass et al.). This lack of activity was due to species-specific differences in the N-terminal domain of ClpX, which are critical for processing the replication clamp loader subunit DnaX in *C. crescentus*. Thus, small differences in ClpX specificity may be particularly critical for specific bacterial species.

In their review on “Functional Diversity of AAA+ Protease Complexes in *Bacillus subtilis*,” Elsholz et al. discuss the functions of several AAA+ proteases in *B. subtilis*, namely: ClpCP, ClpEP, ClpXP, ClpYQ, LonA/B, and FtsH (Elsholz et al.). They discuss how different stress responses control their expression and the phenotypes observed upon deletion of these different proteases. The ability of some of these proteases to control competence, sporulation, motility, and biofilm formation are described. Finally, the authors discuss targeting these proteases for the development of novel antibiotics.

In their review entitled “AAA+ Machines of Protein Destruction in Mycobacteria,” Alhuwaider et al. (Alhuwaider and Dougan) discuss recent advances in determining the structure and function of AAA+ proteases of mycobacteria. These proteases are: ClpXP1P2, ClpC1P1P2, Lon, FtsH, and Mpa. The authors also discuss the Pup-proteasome system (PPS) present in mycobacteria, which is equivalent to the ubiquitin-proteasome system in eukaryotes. Alhuwaider et al. then conclude with a discussion of novel compounds that dysregulate or inhibit the activity of ClpP1P2 and others that dysregulate ClpC1. These compounds have promising activities against mycobacteria.

In a research article entitled “The Copper Efflux Regulator CueR Is Subject to ATP-Dependent Proteolysis in *Escherichia coli*,” Bittner et al. demonstrate that the AAA+ proteases Lon, ClpXP, and ClpAP are responsible for the degradation of *E. coli* CueR, which is a transcription factor that controls the induction of the copper efflux Cue system (Bittner et al.). The authors found that the recognition of CueR by the AAA+ proteases requires the accessible C-terminus of CueR. They conclude that ATP-dependent proteases are required for copper homeostasis in *E. coli*.

Fishovitz et al. carry out a detailed comparison between human and *E. coli* Lon in their research article entitled “Utilization of Mechanistic Enzymology to Evaluate the Significance of ADP Binding to Human Lon Protease” (Fishovitz et al.). By using a detailed mechanistic study, they found that unlike *E. coli* Lon, human Lon has low affinity for ADP despite showing comparable k_cat_ and K_M_-values in the ATPase activity. They propose that human Lon is not regulated by a substrate-promoted ADP/ATP exchange mechanism. These differences between human and *E. coli* Lon might allow the future development of species-specfic Lon inhibitors.

In his review on “Multifunctional Mitochondrial AAA Proteases,” Dr. Glynn discusses the two mitochondrial AAA proteases, i-AAA and m-AAA (Glynn). Both are mitochondrial inner membrane proteins. However, i-AAA projects the ATPase and protease domains into the mitochondrial intermembrane space, while the m-AAA protease projects the catalytic domains into the matrix. The structures of these proteases are discussed as well as their mechanism of function. The proteases can carry out complete substrate degradation, but also can only cleave certain substrates such as for MrpL32 and Atg32.

### ClpB and Hsp104

In their mini-review, “Structural Elements Regulating AAA+ Protein Quality Control Machines,” Chang et al. discuss how pore loop-1, the Inter-Subunit Signaling motif, and the Pre-Sensor I insert motif might contribute to the activity of two Hsp100 disaggregases, bacterial ClpB and yeast Hsp104 (Chang et al.). They propose a model for how these structural elements might enable the AAA+ ATPase cycle to be coupled to substrate translocation across the central channel of ClpB and Hsp104. This process of polypeptide translocation is thought to underpin how ClpB and Hsp104 extract polypeptides from aggregated structures (Chang et al.).

Duran et al. provide a “Comparative Analysis of the Structure and Function of AAA+ Motors ClpA, ClpB, and Hsp104: Common Threads and Disparate Functions” in their review (Duran et al.). They discuss the ability of these three AAA+ proteins (ClpA, ClpB, and Hsp104) to translocate polypeptides through their hexameric complexes. All these proteins have two AAA+ domains and are known to unfold proteins. Importantly, ClpB and Hsp104 are also known to function as disaggregases, while ClpA can form a complex with the ClpP protease. The authors highlight the need to use transient state kinetic methods to examine the kinetic mechanisms of these motor proteins. They describe how the use of such methods allowed them to show that, for example, ClpA translocates polypeptides at about 20 aa s^−1^, while in complex with the ClpP protease, ClpA translocation rate is even higher at about 35 aa s^−1^. The authors also discuss the importance of the Hsp70 chaperone in the function of ClpB/Hsp104,and the observation of species specificity in the interaction between Hsp70 and ClpB/Hsp104.

In their research article entitled “Mutant Analysis Reveals Allosteric Regulation of ClpB Disaggregase,” Franke et al. carry out mutational analysis on the *E. coli* ClpB disaggregase to characterize its allosteric regulation (Franke et al.). ClpB can be divided into an N-terminal domain and two AAA+ domains separated by a helical region termed the M-domain. The authors identify a highly conserved residue in the first AAA+ domain, A328. ClpB-A328V mutant was found to have very high ATPase activity and exhibited cellular toxicity. Unexpectedly, the high ATPase activity of ClpB-A328V was mainly due to the second AAA+ ring as assessed by amide hydrogen exchange mass spectrometry. The authors conclude that A328 is a crucial residue in controlling the ATP hydrolysis in both AAA+ rings of ClpB.

In their research article entitled “Substrate Discrimination by ClpB and Hsp104,” Johnston et al. describe the innate substrate preferences of ClpB and Hsp104 in the absence of the DnaK and Hsp70 chaperone systems (Johnston et al.). They show that substrate specificity is determined by the first AAA+ domain in each protein. They reached this conclusion by testing the two chaperones for their ability to act on several model substrates. They also tested different chimeras of the two chaperones.

In “Hsp78 (78 kDa Heat Shock Protein), a Representative AAA Family Member Found in the Mitochondrial Matrix of *Saccharomyces cerevisiae*,” Abrahão et al. discuss the structure and function of Hsp78 (Abrahao et al.). Hsp78 is the mitochondrial paralogue of Hsp104, which functions in protein disaggregation and reactivation (Abrahao et al.). Curiously, Hsp104 and Hsp78 were lost upon the transition from protozoa to metazoa (Abrahao et al.). However, Abrahao et al. discuss the existence of ANKCLP, which appears alongside Hsp78 and Hsp104 in protozoa and survives the evolutionary transition to metazoa. ANKCLP possesses an AAA+ domain similar to nucleotide-binding domain 2 (NBD2) of Hsp104 and Hsp78, but is otherwise highly divergent. Intriguingly, mutations in ANKCLP cause 3-methylglutaconic aciduria, progressive brain atrophy, intellectual disability, congenital neutropenia, cataracts, and movement disorder in humans (Abrahao et al.).

### p97

In their review, “Structure and Function of p97 and Pex1/6 Type II AAA+ Complexes,” Saffert et al. discuss two different AAA+ complexes that remodel ubiquitinated substrate proteins (Saffert et al.). One function of p97 is to dislocate ubiquitinated substrates from the ER membrane to the proteasome during ER-associated degradation (Saffert et al.). By contrast, Pex1/Pex6 is a heterohexameric motor comprised of alternating Pex1 and Pex6 subunits, which is essential for peroxisome biogenesis and function. Recent cryo-electron microscopy (cryo-EM) structures of p97 and Pex1/6 are discussed and key structural differences are highlighted.

In their review entitled “A Mighty ‘Protein Extractor' of the Cell: Structure and Function of the p97/CDC48 ATPase,” Ye et al. summarize the current knowledge of the structure and function of p97 and its role in several diseases (Ye et al.). p97 has two AAA+ domains connected with a short linker. It also has an N-terminal domain, which mediates its interactions with different adaptor proteins. The authors provide a detailed discussion of the structure of p97 and the effect of nucleotides on its different conformations. These studies are based on using techniques such as EM, X-ray crystallography, and high-speed atomic force microscopy. The authors then discuss the multicellular functions of this highly conserved protein, including its roles in ER-associated protein degradation (ERAD), mitochondria-associated degradation (MAD) by extracting polypeptides from mitochondrial outer membrane, and ribosome-associated degradation (RAD). Finally, Ye et al. provide a summary of p97 mutations leading to several human diseases such as IBMPFD (Inclusion Body Myopathy associated with Paget's disease of the bone and Frontotemporal Dementia)], FALS (familial amyotrophic lateral sclerosis), CMT2Y (Charcot-Marie-Tooth disease, type 2Y), hereditary spastic paraplegias (HSP), Parkinson's disease (PD), and Alzheimer's disease (AD).

In their review on p97 entitled “The Interplay of Cofactor Interactions and Post-translational Modifications in the Regulation of the AAA+ ATPase p97,” Hänzelmann and Schindelin discuss how different cofactors modulate the activity of the p97 ATPase (Hanzelmann and Schindelin). They highlight the fact that the ability of p97 to be involved in a large number of cellular processes is due to the large number of cofactors that interact with this protein. They elucidate three different classes of p97 cofactors, namely: (i) Substrate-recruiting cofactors like UBA-UBX proteins and UFD1-NPL4, (ii) Substrate-processing cofactors like ubiquitin (E3) ligases and deubiquitinases (DUBs), and (iii) Regulatory cofactors like the UBX proteins, which may sequester or recycle p97 hexamers. The authors also discuss the role of post-translational modifications on p97 activity, and on its interactions with its cofactors and substrates.

### AAA+ proteins of the proteasome

In “AAA-ATPases in Protein Degradation,” Yedidi et al. review the activities of Rpt1, Rpt2, Rpt3, Rpt4, Rpt5, and Rpt6, which are the AAA+ ATPases of the eukaryotic proteasome, as well as some of their bacterial relatives such as PAN, Mpa, and VAT (Yedidi et al.). They focus on new technologies to understand how these AAA+ ATPases function by translocating unfolded polypeptides into the proteolytic chamber of the protease (Yedidi et al.). Conformational changes within the AAA+ ring and adjacent chambered protease appear to generate a peristaltic pumping mechanism to deliver substrates for degradation (Yedidi et al.).

In their research article, “The Proteasomal ATPases Use a Slow but Highly Processive Strategy to Unfold Proteins,” Snoberger et al. establish that proteasomal AAA+ proteins employ a low velocity but highly processive motor mechanism to deliver substrates to the proteolytic cavity of the proteasome (Snoberger et al.). This mechanism contrasts with ClpX, which utilizes a high velocity but less processive motor mechanism to deliver substrates to the ClpP protease for degradation. These differences in motor mechanism may have evolved in response to differing demands of their specific clientele.

### Rubisco activases

In their review on “Rubisco Activases: AAA+ Chaperones Adapted to Enzyme Repair” Bhat et al. discuss the unique function of the Rubisco activase (Rca) in remodeling Rubisco (Bhat et al.). Rca is a AAA+ chaperone that is highly conserved in photosynthetic organisms from bacteria to higher plants. Rubisco is Ribulose-1,5-bisphosphate carboxylase/oxygenase enzyme, which is involved in fixing atmospheric CO_2_ during photosynthesis. It is the most abundant protein on earth and is the key enzyme in the synthesis of all organic matter on the planet. However, Rubisco is a poor enzyme and is easily inhibited by side products of its catalytic reactions or by compounds synthesized by some plants under low light conditions. Rca functions to alleviate or “cure” Rubisco from such problematic inhibitions. The authors discuss the structure of Rca from different species and the potential mechanisms of its function.

Dr. Mueller-Cajar provides a review on “The Diverse AAA+ Machines that Repair Inhibited Rubisco Active Sites” (Mueller-Cajar). He discusses the presence of three evolutionarily distinct classes of Rubisco activases (Rcas): (1) green and (2) red-type Rcas that are mostly found in photosynthetic eukaryotes of the green and red plastid lineage, respectively, and (3) CbbQO present in chemoautotrophic bacteria. He discusses the evolution of these activases and their potential use in synthetic biology to enhance Rubisco activity in plants.

### Torsin

In their perspective article, “Torsin ATPases: Harnessing Dynamic Instability for Function,” Chase et al. discuss the Torsins, which are also phylogenetically related to NBD2 of yeast Hsp104 (Chase et al.). Torsins are the only AAA+ ATPases localized inside the ER and connected nuclear envelope (Chase et al.). Intriguingly, mutations in TorsinA cause DYT1 dystonia, a neurological disorder in humans (Chase et al.). Torsins exhibit weak ATPase activity that is augmented via active-site complementation due to co-assembly with specific accessory cofactors LAP1 and LULL1 (Chase et al.). Chase et al. suggest that dynamic assembly and disassembly of Torsin/cofactor complexes play important roles in their function in nuclear trafficking and nuclear-pore complex assembly (Chase et al.).

### Pontin and reptin

In their extensive review on “The Role of Pontin and Reptin in Cellular Physiology and Cancer Etiology,” Mao and Houry discuss the multiple functions of the highly conserved Pontin and Reptin AAA+ ATPases (Mao and Houry). These two proteins typically function together as a complex but can also function independently. The authors highlight the roles of Pontin and Reptin in chromatin remodeling. They also discuss how Pontin and Reptin modulate the transcriptional activities of several proto-oncogenes such as MYC and β-catenin. Mao and Houry elucidate how Pontin and Reptin have been found to be required for the assembly of PIKK signaling complexes as well as telomerase, mitotic spindle, RNA polymerase II, and snoRNPs. The authors conclude with an overview of current efforts aimed at identifying inhibitors of Pontin and Reptin to be developed as novel anti-cancers.

## Concluding remarks

In conclusion, this collection of 21 articles highlights a number of important structural and mechanistic aspects of AAA+ proteins involved in protein repair and degradation. We are excited to see how the field will continue to develop during the ongoing cryo-EM revolution (Egelman, 2016). We anticipate that cryo-EM will enable deeper understanding of how these fascinating molecular machines operate in diverse situations (Gates et al., 2017; Puchades et al., 2017; Ripstein et al., 2017; Zehr et al., 2017).

## Author contributions

All authors listed have made a substantial, direct and intellectual contribution to the work, and approved it for publication.

### Conflict of interest statement

The authors declare that the research was conducted in the absence of any commercial or financial relationships that could be construed as a potential conflict of interest.
